# Expectations of younger patients concerning activities after knee arthroplasty: are we asking the right questions?

**DOI:** 10.1007/s11136-016-1380-9

**Published:** 2016-08-05

**Authors:** Suzanne Witjes, Rutger C. I. van Geenen, Koen L. M. Koenraadt, Cor P. van der Hart, Leendert Blankevoort, Gino M. M. J. Kerkhoffs, P. Paul F. M. Kuijer

**Affiliations:** 1 Department of Orthopaedic Surgery, Foundation FORCE (Foundation for Orthopedic Research Care & Education), Amphia Hospital, Breda, The Netherlands; 2 Department of Orthopaedic Surgery, Academic Medical Center, Amsterdam, The Netherlands; 3 ACES (Academic Center for Evidence - based Sports medicine), Amsterdam, The Netherlands; 4 ACHSS (Amsterdam Collaboration for Health and Safety in Sports), Amsterdam, The Netherlands; 5 ORCA (Orthopaedic Research Center Amsterdam), Amsterdam, The Netherlands; 6 Department of Orthopaedic Surgery, Bergman Clinics, Naarden, The Netherlands; 7 Academic Medical Centre, Coronel Institute of Occupational Health, University of Amsterdam, PO Box 22700, 1100 DE Amsterdam, The Netherlands

**Keywords:** Patient expectations, Knee arthroplasty, Knee replacement, Activity, PROMs

## Abstract

**Purpose:**

Indications for total and unicondylar knee arthroplasty (KA) have expanded to younger patients, in which Patient-Reported Outcome Measures (PROMs) often show ceiling effects. This might be due to higher expectations. Our aims were to explore expectations of younger patients concerning activities in daily life, work and leisure time after KA and to assess to what extent PROMs meet and evaluate these activities of importance.

**Methods:**

Focus groups were performed among osteoarthritis (OA) patients <65 years awaiting KA, in which they indicated what activities they expected to perform better in daily life, work and leisure time after KA. Additionally, 28 activities of daily life, 17 of work and 27 of leisure time were depicted from seven PROMS, which were rated on importance, frequency and bother. A total score, representing motivation for surgery, was also calculated.

**Results:**

Data saturation was reached after six focus groups including 37 patients. Younger OA patients expect to perform better on 16 activities after KA, including high-impact leisure time activities. From the PROMs, daily life and work activities were rated high in both importance and motivation for surgery, but for leisure time activities importance varied highly between patients. All seven PROMs score activities of importance, but no single PROM incorporates all activities rated important.

**Conclusion:**

Younger patients expect to perform better on many activities of daily life, work and leisure time after KA, and often at demanding levels. To measure outcomes of younger patients, we suggest using PROMs that include work and leisure time activities besides daily life activities, in which preferably scored activities can be individualized.

## Introduction

Both total knee arthroplasty (TKA) and uniconcylar knee arthroplasty (UKA) are performed at younger ages than before [1–4] since they are well-accepted, reliable, cost-effective and suitable surgical procedures for end-stage knee osteoarthritis (OA) [5, 6]. Arthroplasty surgery was originally conceived for elderly patients performing activities at low levels. In these early days younger age was even a strict contraindication [7]. Over time indications have expanded to younger and more active patients. Riddle et al. [8] showed that nowadays in the decision-making process for TKA, other factors, such as severity of OA, are considered to play a more important role than age. The volume of TKA-surgeries has increased worldwide, like in the USA up to 200 % over the past decade. Patients younger than 65 years are projected to contribute to the majority of this growth, accounting for more than 55 % of all TKAs in the year 2030 [7, 9]. According to the last annual report of the Dutch Arthroplasty Register (LROI), in The Netherlands, the number of registered KAs has also increased (from 20,558 in 2010 to 26,754 in 2014). In 2014, already 23 % of KAs were performed in patients younger than 60 years old (http://www.lroi.nl/en/home).

Knee arthroplasty (KA) is proven to relieve pain, to return to function and to improve health-related quality of life [10, 11]. Despite these positive effects of KA, still 17–19 % of patients are not satisfied after surgery [12, 13]. Residual symptoms have been identified as an important factor in dissatisfaction, for which mostly no implant-related mechanical failure can be found [14–16]. Chronic pain after KA and other medical, socio-demographic, psychological and biological factors are possible explanatory factors [17, 18], but even when no pain exists and physical functional outcomes are good, still some patients are dissatisfied after KA [15]. Hence, preoperative expectations may also play a role [18–25]. Young age is associated with high preoperative expectations concerning activities after KA [26, 27]. These high preoperative expectations do not predict satisfaction after joint replacement [26], but *fulfilment* of these patient expectations clearly seems to play an important role in patient satisfaction [19]. Current described percentages of fulfilment of expectations after KA range from 100 % satisfaction regarding knee pain alleviation to only about 20 % concerning the ability to participate in sports and leisure activities [28].

In younger patients, mostly excellent results of Patient-Reported Outcome Measures (PROMs) after KA are in contrast with more modest satisfaction scores [2, 9, 29–31]. The Oxford Knee Score (OKS) and EuroQol (EQ-5D) for example, demonstrate good results, but due to lower satisfaction scores there are concerns about existing—so-called—ceiling effects of these PROMs in younger patients. A *ceiling effect* occurs when a measure possesses a distinct upper limit for potential responses and a large percentage of participants score at or near this limit. As a consequence, patients with the highest possible score cannot be distinguished from each other, thus reliability is reduced [32]. An example of a ceiling effect is if more than 15 % of the participants with the same maximum VAS satisfaction score of 100 might have different levels of satisfaction, which cannot be specified by the instrument any further. In that case, the instrument does not have sufficient power to specify different levels of the construct that it is supposed to measure [32, 33]. Regarding the OKS and EQ-5D in younger patients, this would mean that the highest scores are easily reached, although these highest PROM scores do not necessarily reflect the scores of which the younger patient group would be satisfied with [32]. Patients likely expect to perform more, better or different activities than those incorporated in these PROMs, so the ‘content validity’ of these PROMs for this specific patient group is questionable [34]. Therefore, new PROMS were recently developed for younger, active and working KA patients, like the Work, Osteoarthritis or joint-Replacement Questionnaire (WORQ) [33], the broadened New Knee Society Knee Scoring System (New KSS) [35, 36] and the Oxford Knee Score Activity and Participation Questionnaire (OKS-APQ) [37], which is a supplement to the original OKS.

In summary, two gaps in knowledge were encountered, leading to the following two research questions: ‘What are the actual expectations of OA patients younger than 65 years concerning activities in daily life, work and leisure time after KA?’ and ‘To what extent do current PROMs meet and evaluate these activities of importance in younger KA patients younger than 65 years?’ The aim of our study was (1) to identify patient expectations concerning activities after KA and (2) to determine which current PROMs encompass these expectations best.

## Materials and methods

### Study design

#### Focus groups

A focus group study was performed to explore expectations of younger OA patients concerning activities in daily life, work and leisure time after KA. Focus group methodology was used in line with the criteria of the CBO (Dutch Institute for Healthcare Improvement) and the consolidated criteria for reporting qualitative research (COREQ) [38]. Focus group sessions were performed, each with different participants, until data saturation was reached. *Saturation of data* is a term in qualitative research. Theoretically it means that researchers reach a point in their analysis of data that sampling more data will not lead to more information related to their research questions [39]. A moderator (SW) and an administrator (PK) encouraged group interaction to enhance the depth of information obtained. In each focus group, semi-structured discussions were held around three key questions. The research question for daily life activities was: ‘What activities of daily life are you expecting to perform better after KA?’ The same question was formulated for work and leisure time activities. After asking the question, the discussion was started. All participants explored each question until no new items were mentioned anymore. After the focus groups, all participants could rate their satisfaction about whether they were enabled to tell their expectancies regarding activities on a numeric rating scale from 0 (‘not satisfied at all’) to 10 (‘extremely satisfied’). With the permission of the participants, all focus groups were audio recorded. Focus groups were repeated until no new activities were mentioned, meaning that data saturation was reached.

#### Survey

To investigate to what extent PROMs of our interest meet and evaluate activities of importance in younger KA patients, the focus group participants also filled out a survey, in which activities were retrieved from a selection of seven PROMS. We assessed the recommended PROMs of the Dutch Orthopaedic Association (NOV) TKA guideline (2014), which are the Knee injury and Osteoarthritis Outcomes Score (KOOS) [40], Oxford Knee Score (OKS) [41] and EQ-5D [42]. (http://www.orthopeden.org/uploads/IO/nP/IOnPG4j60RcZbdpdkVafrw/Conceptrichtlijn-Totale-Knieprothese.pdf). We also included the activities from two PROMs, which are recently designed for younger TKA patients, OKS-APQ [37] and New KSS [35, 36], and from two Dutch PROMs, typically designed to score activities after KA. These are the Short QUestionnaire to ASsess Health-enhancing physical activity (SQUASH) [43, 44] and Work, Osteoarthritis or joint-Replacement Questionnaire (WORQ) [33].

From these seven clinical scoring systems, 72 activities were extracted, collected in a questionnaire and categorized in activities of daily life (*N* = 28), activities of work (*N* = 17) and activities of leisure time (*N* = 27). Activities were separately scored using Likert scales on importance (‘How important is this activity for you?’ from 0 to 10, in which 0 means not important and 10 means very important), frequency (‘How often do you prefer to perform this activity?’ from 0 to 5, in which 0 means never and 5 means more than once a day) and ‘bother’, i.e. limitation in doing that activity due to knee problems (‘Do you experience knee complaints at the moment while performing this activity?’ from 0 to 10, in which 0 means no bother, and 10 means very much bother).

### Data analysis

To describe the actual expectations of younger OA patients concerning activities in daily life, work and leisure time after KA, a transcription was made from remarks of the writer and completed after listening to the audiotapes of the focus groups. The mentioned activities were analysed and categorized into main activities for daily life, work and leisure time by the moderator (SW) and the administrator (PK) based on consensus. After each focus group, new mentioned forms of activities were added.

To assess the extent of PROMs to meet and evaluate these activities of importance, we presented numbers of responders, importance, frequency and bother scores from the present PROM activities. For each activity, a total score including all three components (importance, frequency and bother) was also calculated according to the Knee Activity Score of Weiss et al. [45]. It was necessary here to transform the scores of importance from 0–10 to 0–5 and the scores of bother from 0–10 to −2 to +2. In the original Weiss score, the factor ‘bother’ is scored positive if there is no pain (+2) after KA, and negative if pain still exists (−2). With our ‘modified’ Weiss score, we represent a ‘motivation for surgery-score’ regarding that specific activity, taking into account importance, frequency and bother. Therefore, we scored pain as positive (+2), resulting in existence of pain to be represented in a higher modified Weiss score, meaning a higher motivation for surgery than when no pain exists (−2). The modified Weiss score (mW score) is therefore defined as mW = 5 + 1/10 [Frequency score x Importance score x Bother score], where frequency score ranges from 0 to 5; 0 is never and 5 is always, importance score ranges from 1 to 5; 1 is not important; and 5 is extremely important, and bother score ranges from −2 to 2; −2 is no pain and 2 is maximum possible pain. The range of the mW score is from 0 to 10, with the highest score representing the highest motivation for surgery.

After scoring each activity separately, for each PROM, we determined the average scores for importance, frequency, bother and mW scores, by calculating the mean scores of all incorporated activities of that specific PROM. Concerning the New KSS, it is important to note that of the 32 activities that can be extrapolated, 15 are of daily life, which all patients need to score. Of incorporated 17 leisure time activities, they are asked to choose the three activities that are most important for them. Further, total scores were based on those three individualized activities.

### Study sample

From May 2014 to February 2015, six focus groups, including 37 participants, were recruited from the surgical waiting list of the Amphia hospital and Bergman Clinics. Inclusion criteria were (1) end-stage OA for which patients were indicated for KA (TKA or UKA), (2) age younger than 65 years and (3) speaking and understanding the Dutch language adequately. The Medical Ethical Committee of the Academic Medical Centre stated that no official approval was required. From the transcriptions of every focus group, we created a list of main activities. After four focus group sessions, of in total 22 different participants, the list consisted of 16 main activities (Table 2). In the subsequent two focus groups, no new main activities were reported, so after six focus groups we concluded that saturation of data was reached. The mean number of participants per focus group was six (SD 2), and mean time span of focus group sessions was 56 min (SD 6) excluding one break of about 15 min.

## Results

### Focus groups

Of the 37 participants, 22 (59 %) were men and 15 (41 %) women. Mean age was 58 years (SD 4 years). Seven of 37 participants were younger than 55 years old. The jobs of the participants were classified according to the International Standard Classification of Occupations, endorsed by the Governing Body of the International Labour Organization in 2008 (ISCO-08) (http://www.ilo.org/public/english/bureau/stat/isco/isco08/). Sports that patients once performed were classified in low, intermediate or high type of impact, according to Vail et al. [46] (Table 1).

**Table 1:** Baseline characteristics of the focus group participants

Variable	Participants (N = 37)
Sex	F = 15
	M = 22
Age	
Mean (SD)	57.7 (4.3)
<55 years	N = 7 (19 %)
ASA classification	N (%)
1	9 (24)
2	23 (62)
3	5 (14)
4	0 (0)
Type of work (ISCO-08)	
Unemployed/early retired	2
Disabled	3
1. Managers	1
2. Professionals	2
3. Technicians & associate professionals	5
4. Clerical support workers	2
5. Service & sales workers	9
6. Skilled agricultural, forestry and fishery workers	1
7. Craft and related trades workers	8
8. Plant & machine operators, and assemblers	2
9. Elementary occupations	2
Type of sports	
Walking	6
Cycling	6
Swimming	4
Running	3
Soccer	3
(Cardio-) fitness/bodybuilding	3
Skiing	4
Hiking	4
Tennis	2
Squash	2
Mountain biking	2
Shooting	1
Rally racing	1
Billiards	1
Sailing	1
Jeu de boules	1
Mountain climbing	1
Horseback riding	1
Golf	1
Ice skating	1
Total	50
Impact of sports*	N (%)
Low-impact	24 (48)
Intermediate-impact	16 (32)
High-impact	10 (20)

* According to Vail et al. [46]

In total, these 37 younger OA patients wished to perform 162 different forms of activities of daily living, work and leisure time better after KA (Table 2). These activities were categorized in 16 subgroups of activities. Mean satisfaction regarding the focus group process was 8.9 (SD 1.1).

**Table 2:** Reported activities (in daily life, work and leisure time) of all six focus groups and categorized in 16 categories of main activities

Activity	Daily life	Work	Leisure time
**Walking**	**X**	**X**	**X**
>10–20 min	X	X	
Several hours	X	X	X
In the woods	X		X
Short distances		X	
Backwards	X	X	
10–20 km	X	X	X
Four-day March			X
To the bulls’ eye			X
In the mountains/slopes			X
With(playful) dog			X
Without brace/device	X	X	
Rapidly		X	X
Uneven ground/in the sand		X	X
Flat ground	X	X	X
City tour			X
Upright	X		
During grocery shopping	X		
With friends			X
On high heels			X
While going out (like museum)			X
During shopping			X
During a whole night shift		X	
*Total walking (22 different ways)*	9	10	15
**Sitting**	**X**	**X**	**X**
>1 h	X	X	
Without changing position		X	
With knees extended	X		
With knees bended	X	X	X
On sport stands			X
In cinema/theatre	X		X
In airplane		X	X
In a car			X
On the toilet	X		
On the knees	X	X	
Working in narrow spaces		X	
During odd jobs (>15 min)	X		
*Total sitting (12 different ways)*	7	6	5
**Standing**	**X**	**X**	**X**
>15 min	X	X	X
While cooking (>4–6 u)	X	X	X
During shooting			X
Without brace or crutches	X		
Billiards/snookering			X
During a party			X
In line at the checkout	X		
During ironing	X		
During a concert			X
While taking a shower	X		
Watching a soccer game			X
While vacuuming	X		
During swimming classes			X
On stairs	X	X	
On a ladder (> 30 min)	X		
Wiping windows	X		
Painting (house)		X	
In public transport	X		
*Total standing (18 different ways)*	11	4	8
**Getting up**	**X**	**X**	
Of a chair	X	X	
Rising of (a low toilet)	X		
After underhand activities	X		
Behind a desk	X	X	
Proper, ‘like a woman’	X		
In and out of bath	X		
Out of bed	X		
Getting of a bicycle	X		
Starting up after sitting	X	X	
*Total getting up (9 different ways)*	9	3	0
**Lifting**	**X**	**X**	
Laundry-basket	X		
Kids	X	X	
(mentally disordered) Patients		X	
Something heavy	X	X	
Groceries	X		
*Total lifting (5 different ways)*	4	3	0
**Cycling**	**X**		**X**
>30–40 km	X		X
A couple of days in a row			X
For grocery shopping	X		
Mountain bike			X
Race (Amstel gold race)			X
With a partner			X
*Total cycling (6 different ways)*	2	0	5
**Driving**	**X**	**X**	**X**
Using (gas) pedals	X		
Stepping in and out	X	X	
In/out of cabin (with jump)		X	
Long distances		X	X
Driving rally			X
On a bus		X	
On a motorcycle			X
*Total driving (7 different ways)*	2	4	3
**Turning and changing movements**	**X**	**X**	**X**
During cooking	X	X	
Making the beds	X	X	
In bed	X		
Pushing a container		X	
Rolling in waterbed	X		
Stepping over something		X	
Stepping in and out the boat			X
*Total turning (7 different ways)*	4	4	1
**Climbing stairs**	**X**	**X**	
Fluently, without asserting feet or grasping hand rail	X		
Drive-in house	X		
With a bucket of water	X	X	
Ladder/cage		X	
Downward	X		
On a scaffold		X	
*Total climbing stairs (6 different ways)*	4	3	0
**Underhand activities**	**X**	**X**	**X**
Household	X		
Painting		X	
Knee bending		X	
Kneeling	X	X	X
To crown	X		
Grabbing something	X		
Gardening	X		X
Tie one’s laces	X		
Compression stockings		X	
Attracting clothes below belt	X		X
*Total underhand activities (10 different ways)*	7	4	3
**Sleeping**	**X**		
Painless	X		
With knee extended	X		
Without waking up	X		
*Total sleeping (3 different ways*	3	0	0
**Holiday activities**			**X**
Sleeping in another bed			X
Sitting in a plane			X
Long drives (car)			X
Making long trips			X
Walking (on slopes/dunes)			X
City tours			X
*Total Holiday activities (6 different ways*	0	0	6
**Grandchildren activities**	**X**		**X**
Lifting	X		
Playing (like ballgames)	X		X
Making (small) trips			X
Babysitting	X		X
Walking behind a buggy			X
Taking a sprint to catch them			X
*Total grandchildren activities (6 different ways*	3	0	5
**Sports and physical activities (PA)**	**X**		**X**
Aerobics/fitness classes			X
(cardio)Fitness/strengthening			X
Bodybuilding/power lifting			X
Jogging			X
With the dog	X		
Sprinting to catch the bus	X		
Sprinting into soccer pitch			X
Along the shore			X
Swimming			X
Skiing			X
Squash			X
Sailing			X
Mountain/wall climbing			X
Jeu de boules			X
Horse riding			X
Tennis			X
Golf (>9 holes)			X
Ice skating (>50 km)			X
Soccer			X
Dancing			X
Polonaise (Carnival)			X
In the disco			X
*Total sports and PA (22 different ways*	2	0	20
**Other (hobbies and) activities**	**X**	**X**	**X**
Sewing (pedals of machine)			X
Going to a terrace			X
Giving soccer training			X
Odd jobs	X	X	X
Game-like working activities		X	
Giving teaching classes		X	
Squatting	X	X	
Working like stay-over dad			X
*Total other activities (8 different ways*	2	4	5
**Awareness and coordination (regarding activities)**	**X**	**X**	**X**
Moving while thinking less	X	X	X
Moving without one hand free	X		
Timing of movements	X	X	X
Quick reactions	X		
Balancing/stability	X	X	X
Walking on high heels			X
Moving without pain	X		
Moving sideward		X	
Concentrating (at work)		X	
No more remarks of colleagues		X	
Keep up a relationship			X
Not always looking for a chair	X		
Participating in work		X	
Keep up with colleagues		X	
Adjustment of work activities		X	
*Total awareness and coordination (15 different ways)*	7	9	5
**Total 16 categories (162 different activities)**	**73**	**54**	**81**

In total, 73 different activities in daily life were mentioned and these were grouped into 15 of the 16 categorized main activities. Noticeable is the fact that for the mentioned activities, different forms and intensities were recalled (Table 2). For example, the subgroup activities standing, walking and getting up were often mentioned (in, respectively, 11, 9 and 9 different circumstances). The activity ‘standing’, for example, was expected to perform better in diverse situations, such as ‘while cooking’, ‘while taking a shower’, ‘on a ladder’, ‘during ironing’ or ‘in line at the checkout’.

Work activities were mentioned in 54 different ways, and these were grouped into 11 of the 16 categorized main activities. Most diverse forms and intensities of mentioned activities concerned the subgroup ‘walking’ (10), varying from ‘walking short distances’ to ‘several hours’ and ‘during a whole night shift’. Awareness and coordination problems, due to their knee problems, were also often mentioned. Examples are ‘moving without thinking’, ‘concentrating’, ‘adjustment of work activities’ and ‘keep up with colleagues’.

Most different activities (81) were mentioned in the leisure time category, and these were grouped into 12 of the 16 categorized main activities. Many hobbies were reported, including a diverse range of sports. All participants mentioned low-impact sports, like walking, swimming, dancing and cycling. However, intensities of these ‘low-impact’ sports varied. ‘Cycling’, for example, is performed both during daily living and in leisure time. In leisure time, they mentioned a wish to cycle longer distances (>30–40 km) or a couple of days in a row. Derived–more extreme–types of cycling were mentioned as well, like the expectation to participate in races and doing challenging mountain bike trips. ‘Walking’ was also mentioned in diverse intensities, varying from a short trip to a ‘Four-day March’ of 30–50 km per day. Hiking and downhill skiing were often-reported intermediate-impact types of sports, besides horse riding, ice skating and mountain climbing. Of the high-impact sports, jogging, playing tennis, playing squash, power lifting and soccer were all mentioned.

### Survey

For the patients in the study, all three categories of activities derived from the PROMs represent a similar motivation for surgery as reflected by the similar scores in the total mW scores between activities of daily life (Table 3), work (Table 4) and leisure time (Table 5).

**Table 3:** Daily life activities of survey; scored importance, frequency, bother and calculated modified Weiss score (ordered in mean mW)

Daily life activities (*N* = 28)	Importance			Frequency			Bother*			mW score		
	*N*	Mean	SD	*N*	Mean	SD	*N*	Mean	SD	*N*	Mean	SD
Kneeling	36	8.1	2.1	37	3.8	1.4	37	8.0	2.5	36	7.4	1.9
Walking, long distance	35	8.7	2.3	36	3.3	1.2	36	8.2	2.5	35	7.1	1.7
Turning	37	8.0	2.8	37	3.8	1.6	36	7.5	2.6	36	7.0	2.1
Crouching	37	7.4	2.4	37	3.7	1.5	37	8.2	2.7	37	7.0	2.3
Walking, uneven ground	36	8.3	2.2	36	3.6	1.3	36	7.6	2.6	36	6.9	2.0
Standing, long time	36	8.3	2.9	35	3.9	1.2	34	7.2	2.7	34	6.9	2.3
Getting up	36	8.5	2.3	37	4.8	0.4	37	6.8	2.8	36	6.6	2.9
Climbing the stairs	37	9.2	1.5	37	4.7	0.7	37	6.9	2.6	37	6.5	2.7
Walking, slopes	36	8.5	2.0	36	3.3	1.3	36	7.6	2.5	36	6.3	2.0
Walking, freely	36	9.3	1.5	36	4.7	0.9	36	6.3	2.7	36	6.2	2.8
Lifting	36	8.4	1.9	36	3.4	0.8	36	6.3	2.6	36	5.9	2.1
Walking, even ground	36	9.1	1.6	36	4.6	0.6	36	5.9	2.6	36	5.9	2.6
Sprinting	31	6.7	2.9	31	1.5	1.6	29	7.4	3.3	29	5.7	1.4
Shopping	34	7.3	2.6	34	2.7	1.2	34	6.1	3.0	34	5.7	2.1
Getting in/out the car	36	9.2	1.5	36	4.3	1.0	36	5.8	3.0	36	5.7	3.0
Walking, short distance	36	9.3	1.3	36	4.2	1.1	36	5.7	2.7	36	5.5	2.6
Groceries	35	8.1	2.4	36	3.6	0.8	36	5.5	2.7	35	5.4	2.2
Bending over	37	7.8	2.2	37	4.4	0.7	37	5.7	3.2	37	5.4	3.1
Getting up ladder	35	7.4	2.7	35	2.7	1.4	33	5.2	3.1	33	5.3	1.9
Walking, with aids	17	5.9	3.7	16	2.5	2.3	14	5.0	3.1	14	5.3	2.1
Moving sideward	37	7.0	2.4	36	4.2	0.7	36	5.4	2.5	36	5.2	2.0
Taking a sidestep	35	7.1	2.4	33	2.9	1.5	34	5.5	2.8	33	5.1	1.7
Household work, heavy	35	7.4	2.4	35	3.1	1.3	33	5.9	2.9	33	5.1	2.1
Getting in/out bath	32	6.5	2.7	32	3	1.6	32	3.9	3.1	32	4.8	1.8
Pulling on socks	37	8.2	2.3	37	4	0.9	37	4.9	3.2	37	4.8	2.9
Using public transport	29	5.3	3.7	27	1.6	1.6	25	2.8	3.3	25	4.7	1.6
Turning in bed	37	8.3	2.2	37	4.4	0.5	37	5.1	3.2	37	4.6	3.0
Household work, light	35	8.3	2.4	35	3.9	1.2	35	4.6	2.7	35	4.5	2.3
**Total**	**35**	**7.9**	**2.3**	**35**	**3.6**	**1.2**	**34**	**6.1**	**2.8**	**34**	**5.8**	**2.3**

*N* number of response, *SD* standard deviation, *mW score* modified Weiss score (from 0 to 10, with highest score representing the highest motivation for surgery)

*‘Bother’ refers to the severity of knee complaints

**Table 4:** Work activities of survey; scored importance, frequency bother and calculated modified Weiss score (ordered in mean mW)

Work activities (*N* = 17)	Importance			Frequency			Bother*			mW score		
	*N*	Mean	SD	*N*	Mean	SD	*N*	Mean	SD	*N*	Mean	SD
Crouching	31	7.4	2.9	31	3.6	1.4	30	7.9	2.8	30	6.9	2.3
Kneeling	30	7.4	3.2	30	3.7	1.3	29	7.3	2.9	29	6.8	2.4
Climbing the stairs	31	8.9	1.8	31	4.6	0.6	31	6.9	3.0	31	6.6	2.9
Walking, on rough terrain	30	7.9	3.0	30	3.6	1.4	30	7.1	3.2	30	6.5	2.5
Standing	31	9.3	1.4	31	4.5	0.6	31	6.9	2.9	31	6.5	3.1
Walking to work	26	7.8	2.6	26	2.9	2.0	25	5.4	3.4	25	5.9	1.9
Heavy work activities	30	7.2	2.9	30	3.2	1.3	30	6.9	3.1	30	5.9	2.0
Pushing/pulling	28	6.6	3.1	28	3.1	1.4	27	6.0	3.3	27	5.8	1.9
Sitting	31	9.4	1.2	31	4.7	0.5	31	6.4	3.0	30	5.7	3.3
Lifting heavy weights	29	6.6	2.8	29	3.0	1.2	28	6.6	2.8	29	5.6	1.9
Climbing	30	6.2	3.3	30	2.4	1.9	28	6.5	3.0	28	5.5	1.6
Light work activities	31	8.9	2.2	31	4.0	1.0	31	5.1	3.0	31	5.3	2.8
Cycling to work	25	8.6	2.8	25	3.0	2.0	24	4.3	3.7	24	5.1	2.8
Working with hands below knee height	25	7.5	3.0	25	3.0	1.8	23	4.4	2.8	23	5.0	1.8
Walking, on level ground	30	9.5	1.2	31	4.6	0.8	31	5.6	2.6	30	4.9	2.8
Operating foot pedals	27	8.5	2.8	27	4.0	1.3	27	4.8	3.1	27	4.8	2.9
Driving	31	8.9	2.6	31	4.1	1.4	31	4.6	2.9	31	4.4	2.8
**Total**	**29**	**8.0**	**2.5**	**29**	**3.6**	**1.3**	**29**	**6.0**	**3.0**	**27**	**5.7**	**2.5**

*N* number of response, *SD* standard deviation, *mW score m*odified Weiss score (from 0 to 10, with highest score representing the highest motivation for surgery)

*‘Bother’ refers to the severity of knee complaints

**Table 5:** Leisure time activities of survey; scored importance, frequency, bother and calculated modified Weiss score (ordered in mean mW)

Leisure time activities (*N* = 27)	Importance			Frequency			Bother*			mW score		
	*N*	Mean	SD	*N*	Mean	SD	*N*	Mean	SD	*N*	Mean	SD
Turning	35	7.9	2.6	31	3.8	1.5	34	6.9	3.1	31	6.4	2.6
Walking	34	7.9	3.1	33	2.8	1.6	32	7.7	2.6	32	6.2	1.4
Jogging	25	6.3	3.1	23	1.8	1.6	24	7.9	3.4	23	6.2	1.4
Jumping	25	5.9	2.9	23	1.8	1.5	24	7.2	3.6	23	5.9	1.3
Participating in sports	33	8.2	2.0	30	2.7	1.2	29	7.1	2.6	29	5.8	1.7
Spinning	28	6.5	3.4	27	2.4	1.5	24	6.0	3.3	24	5.7	1.7
Treadmill running	24	5.5	3.7	23	1.6	1.3	20	6.6	3.4	20	5.6	1.0
Squatting	19	3.7	3.8	17	1.2	1.5	17	4.5	4.5	17	5.6	1.4
Dancing	27	4.8	4.2	26	0.9	1.1	20	5.0	4.1	20	5.4	0.9
Legg press	19	4.5	3.5	17	1.2	1.5	17	4.1	4.2	17	5.4	1.1
Legg extensions	18	4.2	3.5	16	1.0	1.3	16	4.1	4.3	16	5.4	1.1
Gardening	34	7.4	3.2	31	23	0.9	32	5.9	2.7	31	5.4	1.4
Recreational activities	34	8.4	2.0	32	1.4	0.7	33	6.5	2.8	32	5.3	0.9
Interacting with others (like taking care)	36	8.3	2.0	34	2.5	1.0	34	5.4	3.0	34	5.2	1.7
Racket sports	25	4.2	3.5	23	1.0	13	20	4.9	3.9	20	5.2	1.0
Cross trainer	24	4.0	3.8	23	1.4	1.6	18	4.2	3.5	18	5.2	1.0
Stretching/yoga	22	3.4	3.1	21	1.1	1.4	17	3.8	3.6	17	5.2	1.2
Aerobics	22	2.4	2.9	21	1.0	1.6	16	3.6	3.4	16	5.1	0.5
Steps	23	2.5	2.9	22	1.0	1.4	17	3.9	3.9	17	5.1	0.7
Swimming	30	7.0	2.6	28	1.7	1.3	28	5.1	3.5	28	5.1	1.1
Golfing	17	3.2	3.8	16	0.6	1.4	14	3.9	4.0	14	5.0	0.2
Bowling	28	4.2	2.9	26	0.8	0.6	26	4.4	3.3	26	5.0	0.5
Holiday activities	36	9.2	1.9	34	13	0.9	34	5.6	2.3	34	5.0	0.7
Weight lifting	22	1.5	2.2	21	0.7	1.2	15	2.9	3.7	15	4.9	0.3
Cycling	34	9.2	1.5	31	3.7	0.9	33	5.5	3.4	31	4.9	2.8
Family activities	35	9.1	1.7	34	2.3	1.1	34	5.1	2.6	34	4.8	1.4
Sexual activities	33	8.2	2.3	30	3.0	0.7	31	3.9	2.6	30	4.1	1.9
**Total**	**28**	**5.8**	**2.9**	**26**	**1.7**	**1.2**	**24**	**5.2**	**3.4**	**24**	**5.3**	**1.2**

*N* number of response, *SD* standard deviation, *mW score* modified Weiss score (from 0 to 10, with highest score representing the highest motivation for surgery)

*‘Bother’ refers to the severity of knee complaints

With regard to rating daily life activities, overall response rate was high (>90 %). Only the questions regarding walking with aids and using public transport remained unanswered by 20 (54 %) and eight (22 %) participants, respectively, as probably these activities were not applicable to them. The total mean importance of 28 scored daily life activities was 7.9 (SD 2.3). Getting out of a car (9.2), climbing the stairs (9.2) and every type of walking (from 8.3 to 9.3) were activities with highest scores on importance. Total mean mW score was 5.8 (SD 2.3). Restrictions to kneeling, crouching and turning represented the highest motivation for surgery with respect to activities of daily life, indicated by mean mW scores of 7.4, 7.0 and 7.0, respectively (Table 3).

With regard to rating work activities, response rates per activity never exceeded 84 %, as applicability of every activity was dependent on the jobs participants performed. Total mean importance of 17 rated work activities was 8.0 (2.5). Walking on level ground (9.5), sitting (9.4) and standing (9.3) were activities with highest scores on importance. Total mean mW score was 5.7 (SD 2.5). Restrictions to crouching, kneeling and climbing represented the highest motivation for surgery with respect to work activities, indicated by mean mW scores of 6.9, 6.8 and 6.6, respectively (Table 4).

With regard to rating leisure time activities, the response rate concerning general leisure time activities, such as holiday activities, gardening and walking, was high (>90 %), but low for specific activities, like playing golf and leg extensions (<50 %). Total mean importance of 27 leisure time activities was 5.8 (SD 2.9). Cycling (9.2), holiday activities (9.2) and family activities (9.1) were activities with highest scores on importance. Total mean mW score was 5.3 (SD 1.2). Restrictions to turning on the painful knee, walking and jogging represented the highest motivation for surgery with respect to leisure time activities, indicated by mean mW scores of 6.4, 6.3 and 6.2, respectively (Table 5).

The type and number of activities differ per PROM (Table 6). For example, in the EQ-5D, three daily life activities are incorporated, while the WORQ assesses 13 work-related activities. In the New KSS, 18 activities of both daily live and leisure time activities are scored. For every PROM, average scores on importance, frequency, bother and mW score out of the mean scores of incorporated activities were calculated. The mean average importance score of activities of all seven PROMs is 8.2 (SD 1.1). Only the KOOS scored lower than eight on importance, with an average score of 7.9 (SD 0.9). The New KSS presented the highest score on average importance (i.e. 8.7, SD 1.8), and 32 of 37 participants scored a minimum of 3 of 17 leisure time activities of the New KSS. Among these 32 patients, 14 different activities were reported, of which road cycling (23 times, by 72 %), distance walking (17 times, by 53 %), spinning/stationary cycling and gardening (both 14 times, by 44 %) were most frequently mentioned as one of three important leisure time activities.

**Table 6:** PROMs scores: importance, frequency, bother and modified Weiss scores (ordered in mean mW)

PROMs (*N* = 7)	Importance		Frequency		Bother*		mW score	
	Mean	SD	Mean	SD	Mean	SD	Mean	SD
KOOS (13 D, 3 L)	7.9	1.0	3.7	0.9	6.4	1.3	6.0	0.9
OKS (8 D)	8.1	1.3	3.7	1.0	6.1	1.8	6.0	1.0
New KSS (15 D + 3/17 L)	8.7	1.8	3.3	1.0	6.6	2.2	6.0	1.5
OKS–APQ (10 D, 2 L)	8.2	1.1	3.5	0.9	6.2	1.6	5.9	0.9
WORQ (13 W)	8.0	1.2	3.8	0.7	6.2	1.1	5.8	0.8
Equation 5 D (3 D)	8.6	0.6	3.7	1.0	6.0	1.3	5.5	0.9
SQUASH (2 D, 4 W, 3 L)	8.1	0.7	3.2	0.5	5.7	1.1	5.4	0.6
Total	8.2	1.1	3.6	0.9	6.2	1.5	5.8	0.9

*SD* standard deviation, *D* daily life activities, *W* work activities, *L* leisure time activities, *mW score* modified Weiss score (from 0 to 10, with highest score representing the highest motivation for surgery)

*‘Bother’ refers to the severity of knee complaints

The mean average mW score of activities of all seven PROMs is 5.8 (SD 0.9). Although showing the lowest score on average importance, the activities of the KOOS scored a higher than mean average mW score, due to relatively high bother scores of the incorporated activities. Comparing scores of OKS and OKS-APQ shows that adding two extra daily life and two leisure activities in the OKS-APQ resulted in a higher valued importance (from 8.1 to 8.2), but to a decreased mW score (from 6.0 to 5.9). The WORQ is a PROM evaluating only work-related activities with average importance of 8.0 (SD 1.2) and average mW score of 5.8 (SD 0.8).

## Discussion

Meeting patient expectations is of utmost importance to satisfy patients after KA. In order to make a major step forwards in meeting patient expectations, the current study was designed to explore preoperative expectations concerning activities of younger age knee OA patients awaiting KA. Our results show that younger OA patients expect to perform 16 categories of activities better after KA, subdivided in 162 different forms, circumstances and intensities, mostly indicating the wish for an active lifestyle. Of these, 45 % were activities to perform in daily life, 33 % during work and 50 % during leisure time, making the last category most diverse in forms, circumstances and intensities. By the total mean mW scores, the survey showed that activities of daily living, work and leisure time are of similar importance to younger patients in the decision-making process whether or not wanting to proceed with knee arthroplasty. Furthermore, all seven evaluated PROMs incorporate important activities, but not one PROM incorporates *all* activities rated of high importance. Moreover, regarding the large SDs of average importance scores, no PROM incorporates *only* activities rated of high importance.

Our results confirm that younger OA patients expect to perform a diversity of activities better after KA [3, 4]. In line with previous studies, we found that expectations between patients vary, depending on their type of work and preferred life styles [46, 47]. From the literature, we also know that patients’ and surgeons’ expectations can differ. Ghomrawi et al. [48] found that more than 50 % of patients had higher expectations than their surgeons, mostly driven by expectations of high-level activities and extreme ranges of motion. With regard to sports, our focus group study shows that active younger patients expect to perform a diversity of high- and intermediate-impact sports after KA. We recently performed a systematic review concerning return to sports after KA [49]. Although more likely after UKA than after TKA, and possibly with some modifications, we showed that high expectations are not always unrealistic, as some patients were able to return to intermediate and high-impact types of sports [49]. Moreover, also the mentioned low-impact sports varied in intensity, so when not sorted in detail, a higher level of performance can be expected by the patient than by the surgeon [50, 51]. It goes without saying that ‘cycling’ challenging mountain bike trails every week leads to higher impacts to the knee than ‘cycling’ just a short distance on a city bike once in a while.

Understanding the multifactorial patients’ decision-making and their motivation for surgery plays a central role in patient-centred care. In a recent systematic review, ‘expectations’ were mentioned as one of ten important themes [12]. In our study, we further explored these expectations regarding activities. In general, daily life activities and work-related activities appear to be a bit more relevant in the decision-making for surgery than leisure time activities. Concerning the surgeons’ part of the shared decision-making process, Iorio et al. already recognized that for selection of the most suitable implant for the patient, it is important to take into account what activities patients want to perform after KA [52, 53]. Many promising technological innovations in knee implants are being developed for patients who wish to stay more active, like renewed interest in the bicruciate retaining KA [54, 55] and improvements in medial, lateral and patellofemoral unicompartmental KAs [56–58]. To determine which patients may benefit best from KA surgery in general and typically from these presumed technological improvements, exploring expectations of activities in more detail seems to be more essential than ever. Recent studies already revealed that psychological factors, such as patient perception, understanding of illness, depression and anxiety, play an important role in recovery and outcome after knee replacement [59]. Even without taking into account these psychological factors in this study, we now have shown that younger patients expect to perform many different activities after KA. To improve patient satisfaction, we recommend further studies to investigate how to fulfil all these different expectations and to explore what effects these activities have on survivorship of knee implants.

Concerning the choice for a PROM, previous studies showed importance of choosing an outcome measure in which all desired levels of performance could be measured. This implicates that a PROM should not have a considerable ceiling or floor effect [60]. Additionally, we learned from our study that to determine whether a KA is meeting expectations in younger patients, it is crucial to take into account participation in activities of more than only daily life activities. Following the similar motivation for surgery between daily life, work and leisure time activities of the PROMs on a group level and also regarding the outcomes of the focus groups, we are of the opinion that PROMs should address work and leisure time activities in addition to only daily life activities. Moreover, to reflect the personal goals and needs of the patient, preferably these PROMS should be more individualized.

The average PROM scores did not differ much, but the number and types of activities incorporated in the PROMs are highly variable (Table 6). To avoid worldwide creation of even more and larger PROMs, including every possible important activity for younger patients, we suggest choosing a PROM in which incorporated activities can be individualized, like the SQUASH [43] or the New KSS for leisure time activities [35, 36]. Adding weight factors to the incorporated activities might be considered in order to meet patient’s expectation, personal goals and needs even more.

According to the results of our survey, the New KSS scored highest in importance with regard to its activities. Its query of both high-demand activities and three priority activities has already been pointed out as unique in the recent systematic review considering PROMs after TKA [61]. Nakahara et al. [62] also investigated expectations and satisfaction regarding daily life activities of the New KSS. Comparable with the importance scores of our survey, they concluded that daily life activities associated with ‘walking’, ‘climbing up or down stairs’ and ‘getting into and out of a car’, had great impact on meeting expectations and patient satisfaction. Besides the possibility to incorporate activities of individual importance, the New KSS consists of objective surgeon-reported components and patient-reported components regarding expectations and satisfaction as well, making it altogether a committed clinical outcome measure to evaluate younger patients after KA [35, 36]. The Dutch SQUASH is another questionnaire, which can be highly individualized. It contains open questions on habitual activities with respect to daily life, work and leisure time, as well as incorporated standard activities, which are highly valued in our survey [43]. As the SQUASH is especially designed to score physical activities in an adult population and is reproducible, valid and shorter than the New KSS, this questionnaire could also be a useful alternative to quantify activity level. The OKS-APQ seems to be a valuable PROM for younger KA patients as well, as it consists of only eight extra items in addition to the OKS [37]. Those items are four extra—highly valued—activities and four items concerning performance and awareness, such as timing and adjustments of activities, which were also mentioned in our focus groups (Table 2). The WORQ, which was developed to assess physical difficulties in work and is a reliable, valid and responsive questionnaire [33], turned out to consist of activities of importance to younger OA patients and might be used in addition to PROMs, which do not include work-related activities.

A strength of our study is the qualitative nature, as the most appropriate way to collect data to support content validity that adequately reflects the patient perspective. There are no a priori sample size estimations in qualitative research; however, most projects reach data saturation after conducting between four and six focus groups [38, 63]. Also in our study, after four focus groups we could formulate the 16 main activity categories for daily life, work and leisure time. We performed another two focus groups in which no new main activities could be extracted, after which we concluded that data saturation was reached. A consequence of using focus group methodology, however, is that activities reported may be listed only once by 1 of 37 patients. So, by this study, we are not able to quantify importance of all the different activities mentioned. Furthermore, this study is not able to answer the question what activities are important for what type of patients, according to sex, ASA classification or work type. Another strength is that in the survey, patients scored importance of incorporated activities out of some popular and new PROMs. Besides rating importance, by modifying the Knee Activity Score of Weiss et al. [45] and taking into account frequency of performance and bother regarding the activity, we assessed relevance of activities in terms of ‘motivation for surgery’. Lastly, we calculated final outcome scores of the PROMs, with regard to their incorporated activities. A limitation of our study is that the study sample of participants was based on the focus group methodology of data saturation. A study sample of 37 patients was enough to reach data saturation for the focus group study, but this number of participants was probably too small to find clinically relevant outcomes for the additional survey. Because of the relatively small number to perform quantitative analyses, we decided not to statistically test differences of the survey results. So, to assess whether these survey results are representative for the total group of younger KA patients, a population-based study should be performed. Although the studied patient group is small, the participating patients consist of both sexes (*n* = 22 males and *n* = 15 females), and they are working in all areas of the ISCO-08 and participating in a fair amount of different sports, including both low-, intermediate- and high-impact types (Table 1). Thereby, we avoided selection bias in the activities mentioned in the focus groups, and by this diversity, we can still feel comfortable with our results. A second limitation is that seven PROMs were assessed out of 47 currently available knee-scoring systems to assess the success of KA [61]. However, evaluating activities from all existing PROMs seemed not manageable, so we selected the activities in recommended PROMs of the Dutch Orthopaedic Association (NOV) and new PROMs, especially addressing activities for younger patients. From these seven PROMS, we already extracted 72 activities, which took about 30 min time of our patients to score, after the time span of the focus group of approximately one hour. Moreover, one should bear in mind that our summarized PROM score is dependent on the number of activities, as not every PROM incorporates the same amount of activities. Nevertheless, this survey aimed to obtain a first insight whether the assessed activities of daily living, work and leisure time from these PROMs are of value to use for both clinical decision-making and future research concerning activities in this younger group of knee OA patients awaiting KA, in order to increase their satisfaction.

In conclusion, orthopaedic surgeons should realize that younger OA patients have many different expectations of activities of daily life, work and leisure time to perform better after KA. Expectations of leisure time activities varied the most in expected forms, circumstances and intensity and often patients expected activities of ‘low-impact’ to be practiced at more challenging levels. Activities of daily life, work and leisure time from PROMs are valued as similar important according to motivation for surgery. All seven evaluated PROMs score activities, which were rated as important to younger OA patients, but not one PROM covers all activities rated as important. For evaluation of the clinical outcomes of younger KA patients, we suggest choosing knee PROMs that can be individualized and evaluate more than only activities of daily life, including activities of work and leisure time.
